# Swan-Neck Deformity as a Rare Complication of a Fifth Metacarpal Fracture: Surgical Correction Using the Aiache Technique

**DOI:** 10.7759/cureus.92647

**Published:** 2025-09-18

**Authors:** Alhan Castillo Valencia, Luis Yair Nevarez Gamboa, Luis Fernando Ochoa Meza, Tavata Lizbeth Daza Villa, Edgar Morales Flores

**Affiliations:** 1 Surgery, Hospital Regional Universitario de Colima, Colima, MEX; 2 Plastic and Reconstructive Surgery, Hospital Regional Dr. Valentín Gómez Farías - ISSSTE (Instituto de Seguridad y Servicios Sociales de los Trabajadores del Estado), Zapopan, MEX; 3 Surgery, Hospital General Presidente Lázaro Cárdenas del ISSSTE (Instituto de Seguridad y Servicios Sociales de los Trabajadores del Estado), Chihuahua, MEX; 4 Plastic Surgery, Hospital Regional Dr. Valentín Gómez Farías - ISSSTE (Instituto de Seguridad y Servicios Sociales de los Trabajadores del Estado), Zapopan, MEX

**Keywords:** aiache technique, extensor tendon adhesion, hand surgery, metacarpal fracture, swan neck deformity

## Abstract

Metacarpal fractures are among the most common hand injuries, with the fifth metacarpal being the most frequently involved. While many cases can be managed nonoperatively, surgical intervention is required when instability, malrotation, or marked angulation is present. We present an unusual complication of swan-neck deformity following fixation of a fifth metacarpal shaft fracture with a dorsal titanium miniplate in a 16-year-old male patient. Initial fixation provided stability; however, progressive deformity developed, characterized by hyperextension of the proximal interphalangeal joint, with compensatory flexion of the distal interphalangeal joint - likely related to extensor tendon imbalance from scarring or hardware placement.

Due to the functional and cosmetic implications, corrective surgery using the Aiache technique was performed. Postoperative follow-up demonstrated bone consolidation, improved range of motion, pain relief, and restoration of hand function.

This case highlights the importance of recognizing rare postoperative complications after dorsal plating of metacarpal fractures. Although swan-neck deformity is uncommon, it can result in significant disability if untreated. The Aiache technique offers a reliable reconstructive solution by directly correcting extensor mechanism imbalance and restoring digital alignment, underscoring the need for careful postoperative monitoring to preserve optimal hand biomechanics.

## Introduction

Metacarpal fractures rank as the second most common type of hand fracture, with an estimated incidence of 8.4 cases per 10,000 person-years [1]. The fifth metacarpal is the most frequently fractured metacarpal. Fractures can be categorized by location as involving the head, neck, shaft, or base of the bone. In terms of morphology, the fracture pattern may be described as transverse, short oblique, long oblique, or comminuted [2].

These fractures typically exhibit palmar angulation due to the deforming forces exerted by the interosseous muscles and the comminution of the volar cortex, which results in instability. A sagittal angulation exceeding 30°, or shortening greater than 5 mm, can alter the biomechanics of the flexor apparatus and potentially lead to functional impairment [3].

The Aiache technique, originally described as a method for correcting swan-neck deformities, consists of advancing the lateral bands volarly to restore balance between flexion and extension forces at the proximal interphalangeal (PIP) joint. This approach directly addresses the extensor mechanism imbalance that characterizes the deformity, often secondary to tendon adhesions, capsular alterations, or postoperative scarring. In the context of our patient, who developed a swan-neck deformity after fixation of a fifth metacarpal fracture with titanium miniplates, the Aiache technique becomes particularly relevant, as it offers a surgical solution aimed at reestablishing functional alignment, preventing further impairment of hand biomechanics, and improving overall hand function [4].

We present the case of a fifth metacarpal fracture that was initially managed with titanium miniplates, followed by the subsequent development of a swan-neck deformity in the same finger. 

## Case presentation

**Figure 1:**
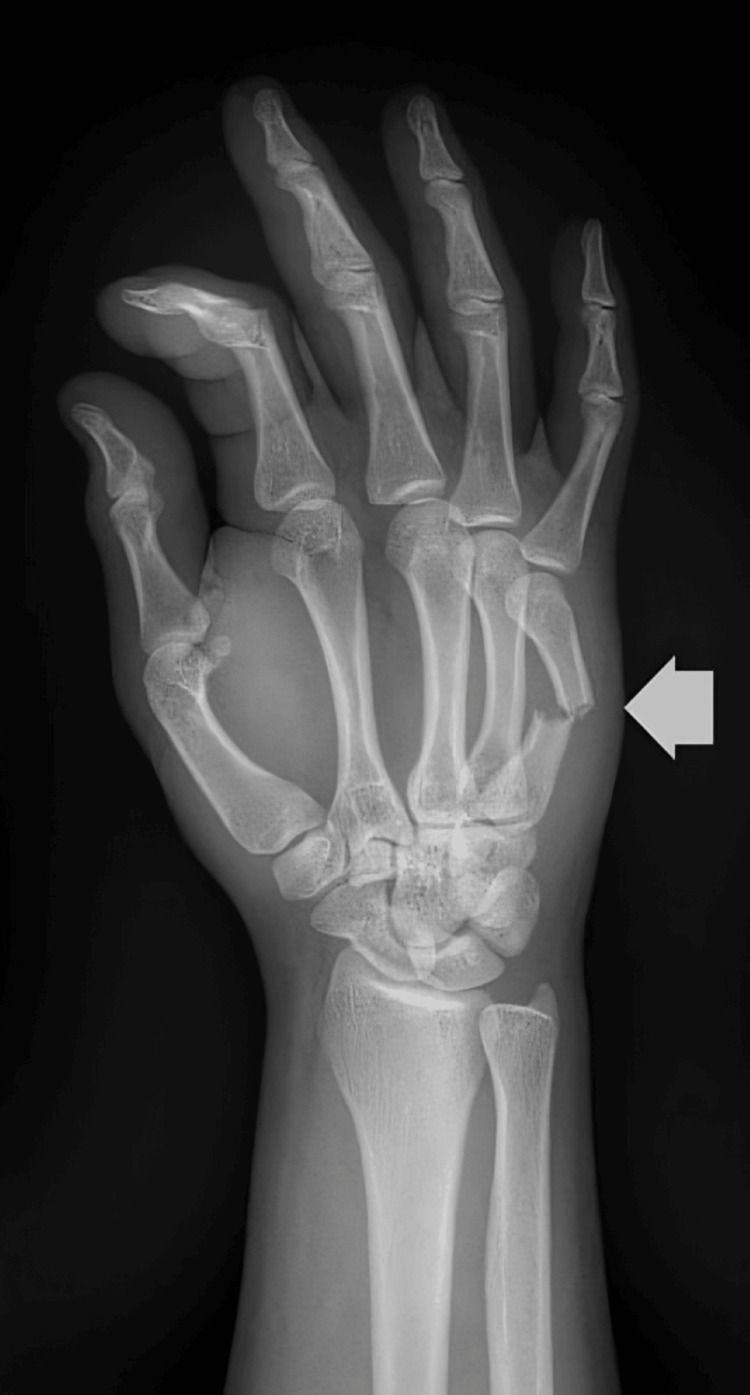
Radiographic image of the right hand. The white arrow highlights a fracture of the shaft of the fifth metacarpal.

A 16-year-old male patient presented after striking a wall with his right hand, experiencing pain, swelling, and limited mobility of the fifth metacarpophalangeal joint. The patient had no relevant past medical history. Initial self-management with cold compresses and analgesics did not result in improvement. Right-hand radiographs, as seen in Figure 1, revealed a fracture of the shaft of the fifth metacarpal.

**Figure 2:**
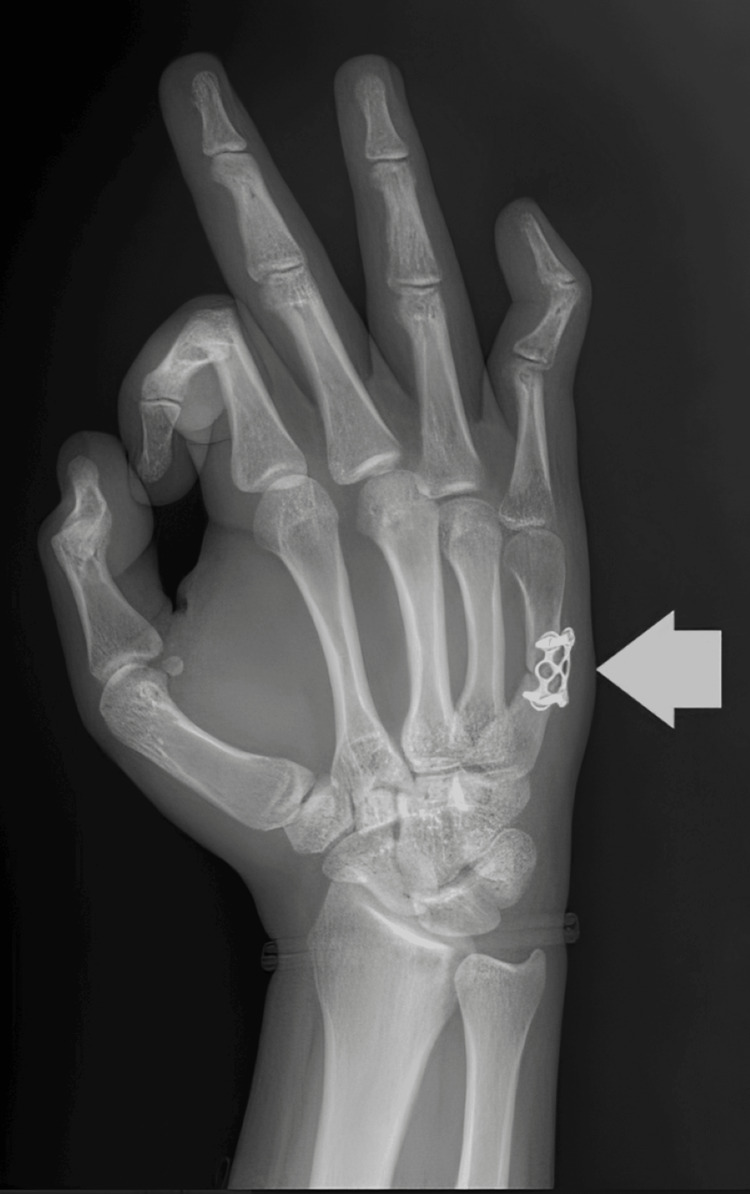
Radiograph image of the right hand. The white arrow indicates a titanium miniplate fixed to the shaft of the fifth metacarpal.

At the follow-up visit, surgical treatment was proposed, and the associated risks were explained. The patient subsequently underwent open reduction and internal fixation with a titanium miniplate (Figure 2). 

**Figure 3:**
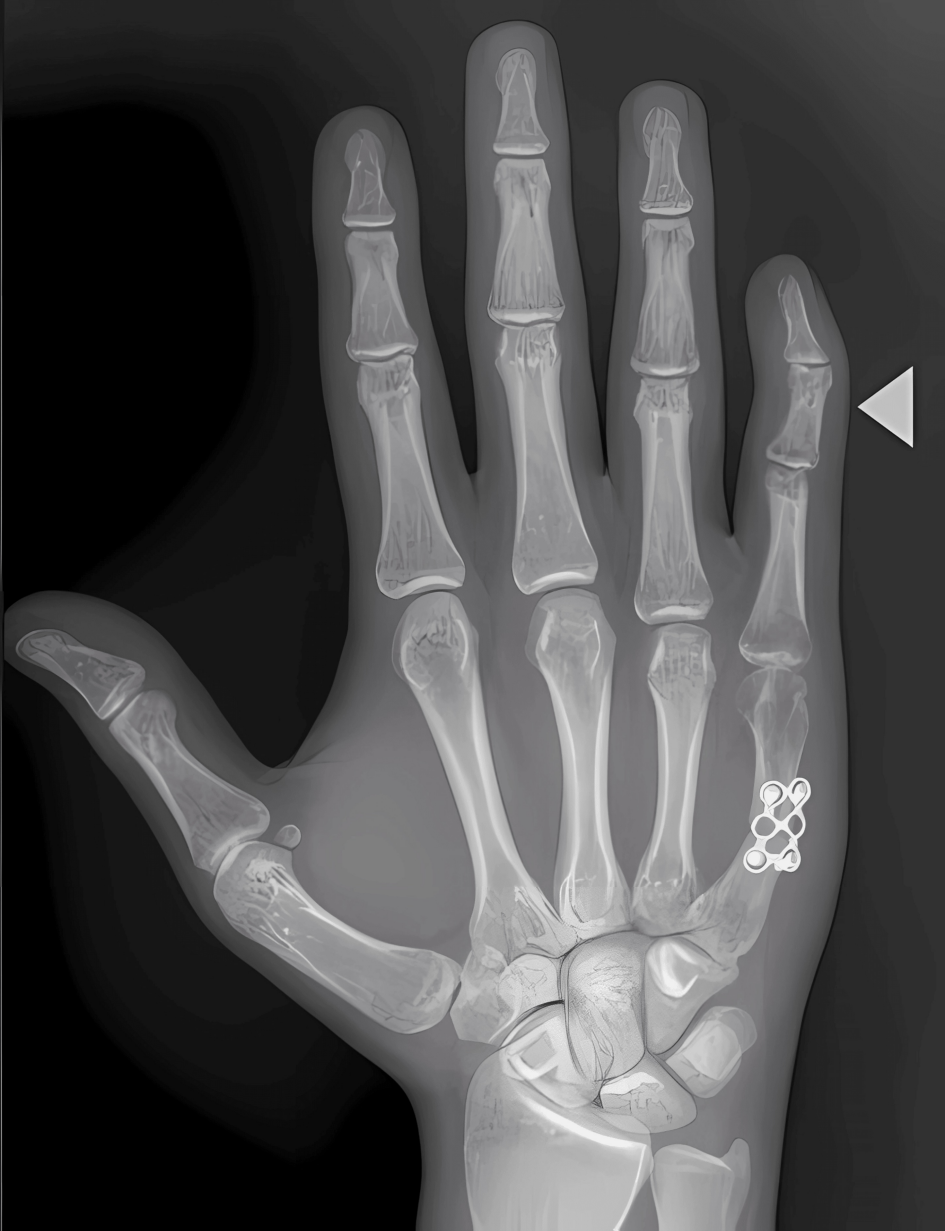
Radiograph of the right hand. The white arrowhead demonstrates a swan-neck-type deformity.

Despite initial adequate fixation, the patient progressively developed a swan-neck-type deformity in the same digit over the following weeks (Figure 3).

**Figure 4:**
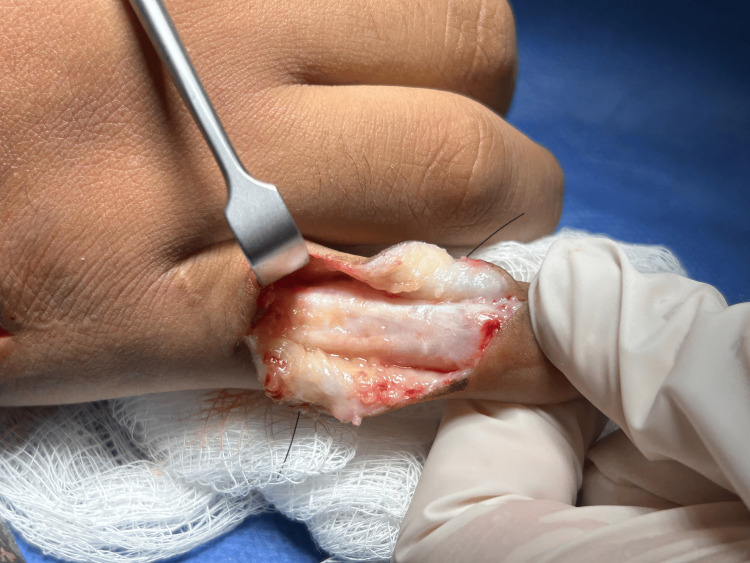
Intraoperative image showing the dorsal incision, with isolation of the extensor tendon lateral bands and controlled release of adhesions or fibrosis prior to their transfer to the volar aspect.

**Figure 5:**
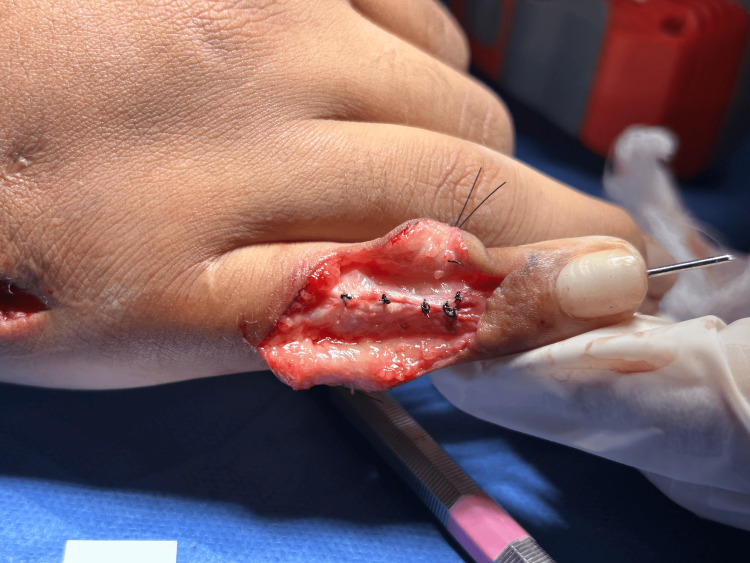
Intraoperative image demonstrating the transfer of the lateral bands to the volar aspect of the proximal interphalangeal joint, secured in their new volar position with non-absorbable sutures.

Given the functional and aesthetic implications, surgical management with the Aiache technique was performed with a satisfactory outcome (Figures 4-5).

**Figure 6:**
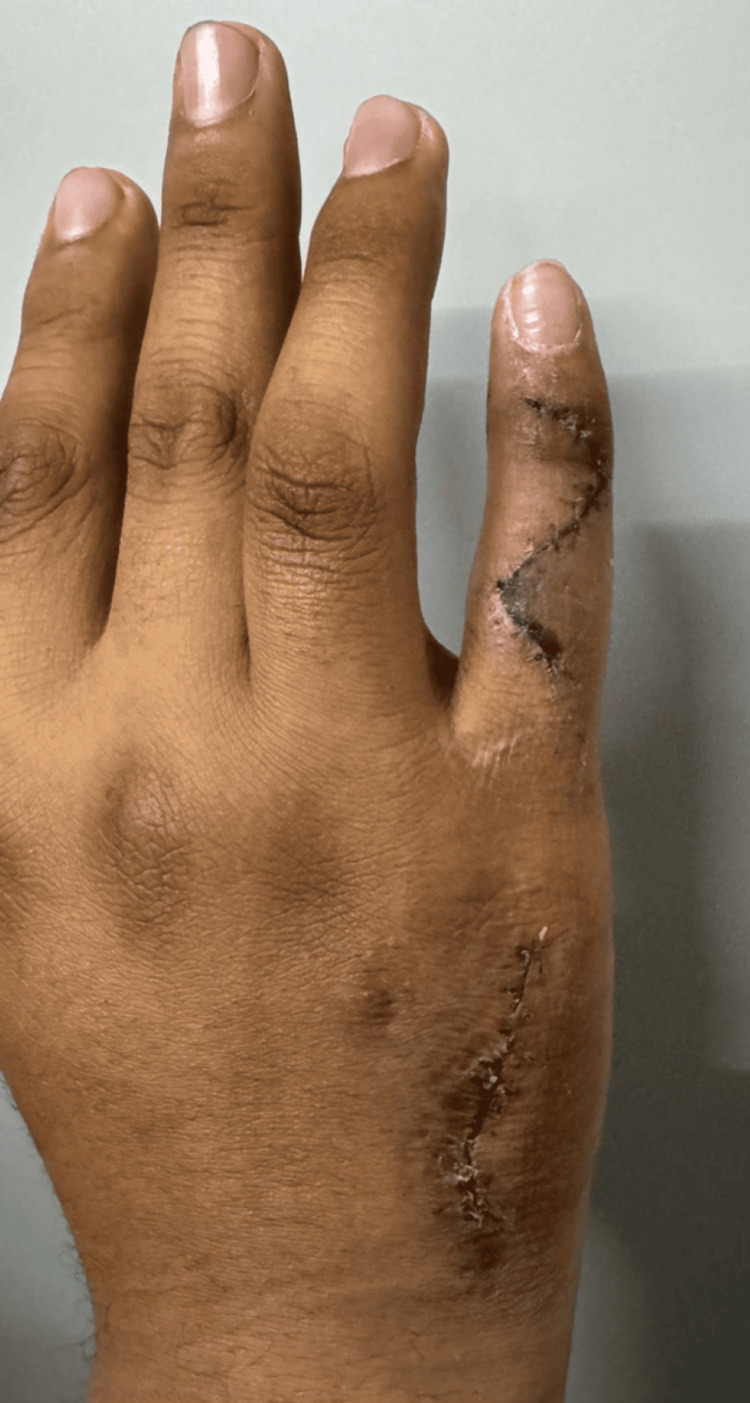
Clinical result at seven days postoperatively, showing appropriate wound healing and satisfactory early functional recovery.

As this case is recent, a definitive conclusion cannot yet be drawn; however, at the most recent follow-up visit, the patient reported improvement, with minimal pain, and expressed satisfaction with the aesthetic outcome (Figure 6).

## Discussion

We present the case of a male patient who arrived at our Emergency Department after striking a wall with his right hand, experiencing deformity and severe pain. A diagnosis of fracture of the shaft of the right fifth metacarpal was made. Following fixation with a titanium miniplate, he was discharged home without complications. What makes this case particularly unusual is the progressive development of a swan-neck-type deformity over the following weeks. 

Most metacarpal fractures are amenable to nonoperative management, and in many cases, accepting a mild degree of deformity is considered preferable to surgical intervention. Fifth metacarpal fractures, in particular, tend to demonstrate favorable outcomes with conservative treatment [5].

Operative management is primarily reserved for displaced intra-articular fractures, extra-articular fractures with over 30° of angulation post-reduction, cases associated with polytrauma, significant soft tissue compromise, unstable open injuries, segmental bone loss, or multiple fractures of the hand and wrist [6]. In the setting of isolated, closed metacarpal fractures, surgical intervention is warranted when closed reduction fails, particularly in the presence of residual malrotation or marked shortening [6]. 

In the present case, the patient sustained a diaphyseal fracture of the fifth metacarpal with a simple transverse pattern, according to the Orthopaedic Trauma Association (OTA) classification [7]. Management options for metacarpal fractures vary from nonoperative treatment to surgical techniques, which include Kirschner wire (KW) fixation and intramedullary compression screw (IS) fixation [8].

The selection of a specific surgical technique remains a subject of considerable discussion and is influenced by multiple factors, including the fracture’s location, presence of rotational deformity, comminution, stability, axial shortening, and overall bone alignment [9]. Patient-related considerations, the surgeon’s expertise, and implant availability also play an important role. Both KW fixation and IS fixation have demonstrated consistent reliability in the treatment of metacarpal neck and shaft fractures [9].

Among the postoperative complications reported in the literature are minor extensor lag, contractures, and major extensor lag. Serious complications are uncommon, including nonunion, infection, and tendon rupture [10].

Swan-neck deformity of the little finger represents an uncommon postoperative complication, characterized by hyperextension of the PIP joint, accompanied by flexion of the distal interphalangeal (DIP) joint [10]. The underlying etiology is multifactorial. Reported mechanisms include injury or adhesion of the extensor digiti minimi tendon, compromise of the volar plate of the PIP joint, and intrinsic imbalance between the lumbrical and interosseous muscles relative to the deep flexor tendons. Notably, dorsal miniplate fixation has been associated with a higher risk of fibrosis and restriction of tendon excursion due to its close anatomical relationship with the extensor apparatus [10].

Diagnosis is essentially clinical, requiring careful assessment of both active and passive range of motion and may be supported by dynamic ultrasonography to detect tendon adhesions. Treatment should be tailored to the severity of the deformity: early physiotherapy and dynamic splinting are often effective in mild or early cases, whereas persistent or functionally limiting deformities may necessitate surgical management, including adhesion release or tendon-rebalancing procedures [10].

The Aiache technique is a surgical procedure designed to correct swan-neck deformity by volarly transposing the lateral bands of the extensor apparatus at the PIP joint. This maneuver restores the normal balance between flexion and extension forces, counteracting the hyperextension at the PIP and the compensatory flexion at the DIP joint. 

By reorienting the lateral bands, the technique addresses the extensor mechanism imbalance that may result from tendon adhesions, scarring, or volar plate insufficiency. In the present case, where the patient developed a progressive swan-neck deformity of the little finger after dorsal fixation with a titanium miniplate, the Aiache technique represents a valuable reconstructive option to reestablish digital alignment, improve tendon gliding, and preserve functional hand biomechanics [10].

Our patient demonstrated satisfactory postoperative progression during outpatient follow-up. Clinical evaluations revealed adequate pain control, absence of infection, and progressive improvement in hand mobility. Serial radiographs confirmed proper alignment and consolidation of the fifth metacarpal fracture, without evidence of hardware failure or secondary displacement. These findings highlight the effectiveness of close clinical and radiographic surveillance in ensuring optimal recovery. 

## Conclusions

This case highlights an uncommon complication of a fifth metacarpal shaft fracture, with the progressive development of a swan-neck deformity despite appropriate fixation. Early recognition of this rare presentation is crucial to prevent functional impairment. The Aiache technique proved to be a reliable surgical option, restoring alignment and function with satisfactory results. This report emphasizes the importance of meticulous surgical planning, close follow-up, and timely intervention when atypical deformities arise after otherwise standard fracture management. 
